# Assessment of the Effectiveness of Fascial Manipulation in Patients with Degenerative Disc Disease of the Lumbosacral Spine

**DOI:** 10.3390/life15010033

**Published:** 2024-12-30

**Authors:** Anna Mikołajczyk-Kocięcka, Marek Kocięcki, Lech Cyryłowski, Aleksandra Szylińska, Paweł Rynio, Magdalena Gębska, Ewelina Szuba, Jarosław Kaźmierczak

**Affiliations:** 1 Department of Cardiology, Pomeranian Medical University in Szczecin, 70-204 Szczecin, Poland; jaroslaw.kazmierczak@pum.edu.pl; 2 CM Luxmed, 71-140 Szczecin, Poland; kocieckimarek@o2.pl; 3 Affidea Zachodniopomorskie Centrum Medyczne, 70-550 Szczecin, Poland; blecyr@hotmail.com; 4 Department of Cardiac Surgery, Pomeranian Medical University in Szczecin, 70-111 Szczecin, Poland; aleksandra.szylinska@pum.edu.pl; 5 Department of Vascular Surgery, Pomeranian Medical University in Szczecin, 70-111 Szczecin, Poland; pawel.rynio@pum.edu.pl; 6 Department of Rehabilitation Musculoskeletal System, Pomeranian Medical University in Szczecin, 70-204 Szczecin, Poland; magdalena.gebska@pum.edu.pl; 7 Department of Humanities and Occupational Therapy, Pomeranian Medical University in Szczecin, 71-103 Szczecin, Poland; ewelinaszuba01@gmail.com

**Keywords:** fascia, low back pain, physiotherapy

## Abstract

**Background:** The aim of this study was to evaluate the effectiveness of Fascial Manipulation in patients with disc herniations of the lumbar spine confirmed by magnetic resonance imaging. **Material and Methods:** This study included 69 patients with intervertebral disc damage of the lumbar spine, as confirmed by magnetic resonance imaging. Patients were divided into two groups: a study group and a control group. The control group (C) was treated conservatively with treatments such as interference currents, diadynamic currents, tens currents, galvanization, Sollux lamps, local cryotherapy, magnetic fields, therapeutic massages of the spine, and mobility exercises. The study group (S) was subjected to therapy using Fascial Manipulation, which included two treatments—the first on the day the patient reported for the study and the second a week later. Patients for this study were selected at random. **Results:** This study presents a statistically significant difference between the treatment effects, as assessed by the degree of pain (*p* < 0.001) and mobility limitation (*p* < 0.001), as well as the presence of stretch symptoms (*p* < 0.001): all three parameters improved significantly more in the study group compared to the control group. **Conclusion:** Fascial Manipulation is an effective method for treating pain in patients with disc herniations of the lumbar spine; in addition to reducing pain, it improves the range of motion and the results of SLR and PKB tests. In lumbar spine pain in disc herniations, treatment using Fascial Manipulation is definitely more effective than treatment according to the traditional physiotherapy regimen.

## 1. Introduction

Low back pain (LBP) is an ailment that affects up to 90% of the population [1,2]. It accounts for 80% of all cases of back discomfort [3]. The diagnosis of its causes and the implementation of appropriate treatment are problematic due to the fact that back pain can be a symptom of a disease originating in organs and tissues distant from the spine [4]. This is confirmed by the fact that only 15% of LBP cases are identified as a specific condition, such as spinal canal stenosis. The remaining 85% of cases are identified as nonspecific low back pain, which often has no identifiable cause and no changes in radiology imaging [5,6]. In nonspecific back pain, LBP may be only a symptom and most often has the features of receptor pain or neurogenic pain [7].

The existence of disc herniations that significantly narrow the spinal canal or cause pressure on the spinal nerve roots in the lumbar spine is frequently an indication for surgery. However, the removal of disc material compressing nerve structures does not always result in an improvement in the patient’s condition, indicating the existence of a non-disc cause of root or meningeal symptoms. This is confirmed by the frequent inconsistency between patients’ symptoms and signs and MRI images. This inconsistency is referred to as nonspecific lumbar spine pain or pseudo-root pain [8]. Clinically, this means that the dysfunction or pain of some structure of the human body can be caused by the prolonged action of a stress factor on another part of the body, sometimes quite distant.

The aforementioned mechanism can be described based on the theory of tensegrity (or tensional integrity). In this approach, the fascia needs compressive components and tensile components to maintain morphological and mechanical equilibrium [9]. The compressive components are bones, which also have some ability to transmit tension. The tensile components include the musculo-fascial system, body cavities such as the pleura, peritoneum, pericardium, and mesentery, and, at the micro level, cells and vacuoles. Compressive components provide support for the tensile elements and protect the entire structure from collapse. The tensile elements keep the compression elements in proper alignment and at proper distances from each other [10].

One of the therapeutic methods employing tensegrity theory is Fascial Manipulation. In this method, the fascia is assigned specific functions and is treated as a component coordinating the motor units grouped in a given musculo-fascial unit (MFU), unifying unidirectional muscle chains (MFU sequences), and connecting body segments (joints) by means of retinacula (MFU spirals) [11].

A musculo-fascial unit consists of fascia and a group of motor units that move a particular body segment in a specific direction. There are six unidirectional MFUs coordinating motion at each joint. According to Stecco, the human body comprises 84 MFUs. Motion in each of these units is controlled by short-vector monoarticular components and long-vector biarticular components (e.g., the biceps and triceps of the arm). There are also shorter vectors formed by single muscle fibers. These are arranged between the two main vectors, giving better control of the entire segment. Thanks to this organization, the continuity and possible coordination between vectors are maintained. The entire structure constitutes a continuity with the tendons and is responsible for the distribution of muscle force. Thanks to the continuity of the connective tissue, it is possible to transmit contractions from the muscle spindles located in the deepest layers to the superficial layers and transmit the passive tension of the fascia in the opposite direction. The tension in this system does not diffuse but is coordinated by a specific center of coordination (CC) [12].

The centers of coordination are responsible for regulating and coordinating the unidirectional muscle fibers of a single MFU by combining the activity of selected motor units to perform a specific movement. CCs have the ability to adapt to the traction of muscle spindles, making the synchronization of muscle fibers by CCs more efficient than that occurring through afferent impulses from free nerve endings.

Muscle spindles attach to the endomysium and tighten the entire fascial structure. The tension of the fascia is concentrated at a single point (CC). When the muscle spindle fibers contract, the annulospiral endings are stimulated. Type Ia and Ib (afferent) nerve fibers attaching to these endings send impulses to the spinal cord. Only then, via alpha fibers, can the contraction phase begin. This activity is imperceptible, but its disruption is manifested as joint pain [13].

Any fascial disorders such as strain, chronic inflammation, metabolic disorders, and trauma can cause densification of the fascia, as well as changes in spatial orientation and an increase in the number of collagen fibers within the fascia. The thickened CC loses its ability to adapt to the tensions of the muscle spindles, resulting in the contraction of only a portion of the MFU muscle fibers, thus distorting the forces acting on the joint [14].

With balanced fascial tension on both sides of the body, only periodic connective tissue hypertrophy occurs in the area of injury, but without decompensation. Tissue adhesion and the chaotic arrangement of collagen fibers can be a factor that promotes repeated inflammation. On one hand, this causes the excessive production of fibronectin and, on the other hand, the pain accompanying the inflammation forces a non-physiological posture or even immobilization [8].

Fascial densification is not removed in the process of self-healing because the body cannot recognize the excess number of collagen fibers. The thickening of the matrix can be palpated as a thickening, which is often painful. The modification of the structure of such a lesion can only be removed by external action (such as Fascial Manipulation) [15].

In addition to pain in the perceptual center, an altered CC (fascial center of coordination) can also cause a blockage of mobility in the joint. In the short term after the blockage, mobilization of the joint is useful. By unblocking the joint, the nociceptive afferent impulse is reduced, thus reducing the increased muscle tension. The situation is different in the case of chronic CC densification, where you should directly work the thickened CC [16].

The manipulation time should be long enough to generate the appropriate temperature through friction. Increasing the temperature at the site of densification modifies the consistency of the basic substance and starts an inflammatory process that lasts several days. Excess fibronectin is removed (thanks to the temperature elevation) and then the healing process restores the normal elasticity of the fascia. In order for the therapy to be effective, a therapist should not focus on only one point, but also take into account all postural decompensation, since only the tension balance of the fascia determines the orderly arrangement of new collagen fibers. Thus, the question arises of to what extent such manipulation can be effective in alleviating discomfort in patients with disc herniations, and what factors may determine its effectiveness—issues that are the primary subject matter of this study.

The purpose of the study is to evaluate the effectiveness of Fascial Manipulation in patients with disc herniations of the lumbar spine confirmed by magnetic resonance imaging.

## 2. Study Material and Methodology

### 2.1. Study Population

This study was carried out at the Koremed physiotherapy office in Szczecin between 1 February 2019 and 14 February 2020. This study included 69 patients with intervertebral disc damage of the lumbar spine, as confirmed by magnetic resonance imaging. The types of intervertebral disc damage included features of degenerative disc disease, namely dehydration and intradural displacement in the form of a fibrous annular protrusion or nucleus pulposus herniation.

### 2.2. Exclusion Criteria

Subjects with contraindications to therapy such as incomplete bone fusion after fractures, cancerous tumor, acute inflammation, pony tail syndrome, fresh injuries with contusions, and qualification for urgent surgery were rejected during study eligibility. Exclusion criteria also included rheumatoid arthritis (RA), ankylosing spondylitis (AS), collagenosis, paresis and paralysis after hemorrhagic or ischemic strokes, multiple sclerosis, and other extrapyramidal syndromes.

### 2.3. Ethical Issues

This study was performed in accordance with the Declaration of Helsinki. It received a waiver from the Bioethical Committee of the Pomeranian Medical University (decision no. KB-0012/15/01/19).

### 2.4. Data Collection

Prior to therapy, all patients underwent an orthopedic examination using the Cyriax method which included active examination, passive examination, and the determination of the neurological status (resistance tests, sensory and reflex tests, and the straight leg raise (SLR) and prone knee bend (PKB) tests). Magnetic resonance imaging was performed according to a routine protocol for examination of the lumbosacral spine with fast spin echo (FSE) sequences in T1-weighted, T2-weighted, and STIR images in the sagittal view, T2-weighted images in transverse projection, and in some cases, T2-weighted images in the frontal view.

Patients were divided into 2 groups. The study group (S) was subjected to therapy using Fascial Manipulation, which included two treatments—the first on the day the patient reported for the study and the second a week later. The control group (K) was treated conservatively using procedures such as interferential currents, diadynamic currents, TENS currents, galvanization, Sollux lamps, local cryotherapy, magnetic field therapy, therapeutic spine massages, and mobility exercises. The mobility exercises were conducted 3 times a week for 45 min each session, including breathing exercises, isometric exercises, and exercises shaping the muscular corset—strengthening the muscles of the lumbopelvic region—as well as learning proper movement patterns, correct posture, and body mechanics. A classic therapeutic massage was performed every other day, lasting 25 min. Physiotherapy treatments were carried out daily depending on the patient’s response: diadynamic currents or TENS currents, galvanization, magnetic field therapy, Sollux lamps, or local cryotherapy. The parameters of the physiotherapy treatments were based on pre-programmed settings found in the devices used for physical therapy.

The goal of the therapy was to eliminate densification, i.e., to restore tissue elasticity. The treatment of a selected CC (coordination center) involved massaging it in all directions using the fingertips, the elbow, or the flat application of the second phalanges of the fingers. The treatment of a single CC was conducted until

- a reduction in pain by about 50%;
- tissue mobility was regained;
- or for no longer than 10 min.

After each CC treatment, a movement verification of the segment subjected to therapy was performed. The treatment results are recorded in the Results section as follows:

- 0—no change;
- (-)—worsening;
- ×—improvement of CC by about 50%;
- ××—improvement in the MO VE (movement) of the segment in all planes or improvement of the MO VE of all segments in one plane;
- ×××—improvement in the MO VE of all segments in all planes.

Since fascial tension changes caused by therapy may appear only after five or six days, the next therapy session took place a week later. If the patient’s symptoms had disappeared, the therapy using Fascial Manipulation was complete. However, if the patient’s condition had not fully improved, another session was conducted following the pattern of the first session. Sometimes, the elimination of one compensation causes the emergence of another. This occurs when a compensation created by the body to balance an unstable posture is removed, resulting in a decompensation of balance. Therefore, if the patient’s symptoms have not decreased, a re-examination of the patient should be carried out and the hypothesis should be adjusted.

In addition, we collected data regarding age, sex, type of intervertebral disc damage, number of damaged discs, degree of intervertebral disc dehydration, cross-sectional area of the spinal canal (canal stenosis or lack thereof), myeloid degenerative reaction in the marginal (lower or upper) parts of the lumbar vertebral bodies adjacent to the endplates, and the compression on the spinal nerve roots or the lack of compression.

To assess the degree of intervertebral disc dehydration, the five-grade Pfirrmann scale was used, taking into account the disc morphology in T2-weighted MRI:

− Grade I: Normal disc—its structure is homogeneous, with a high signal (bright), i.e., hyperintense, and the normal height of the disc is preserved;
− Grade II: The structure of the disc is somewhat heterogeneous, still with a high signal, with a clear signal difference between the nucleus and the annulus, and the normal height of the disc is preserved;
− Grade III: The disc structure is heterogeneous, with intermediate signal intensity. The difference between the nucleus and the annulus is obliterated. The disc height is normal or slightly reduced;
− Grade IV: The structure of the disc is heterogeneous, with a low signal (dark); the distinction between the nucleus and the annulus disappears. The height of the disc is normal or moderately reduced;
− Grade V: The disc structure is heterogeneous, with a markedly low signal; the differentiation of the signal between the nucleus and the annulus disappears. The disc space is collapsed [17].

Central spinal canal stenosis was diagnosed when its anteroposterior dimension on transverse sections at the level of the disc was less than 13 mm; a dimension of 10–12 mm corresponds to so-called relative stenosis, while less than 10 mm is absolute stenosis. This is quite common, but is not the only criterion of canal stenosis [18]. Degenerative lesions of the vertebral bodies were evaluated on the basis of the bone marrow signal in MRI using the Modica scale [19]:

− Type 1 lesions are hypointense in T1-images and hyperintense in T2-images, which denotes inflammation;
− Type 2 lesions are hyperintense in T1-images and isointense or slightly hyperintense in T2-images, which is interpreted as fatty transformation of normal red marrow due to ischemia;
− Type 3 lesions are hypointense in both T1- and T2-images and represent subchondral sclerosis and bone fibrosis [20]. The normal anatomic image is often referred to as Modic 0.5.

Type 1 lesions are probably of inflammatory origin and appear to be strongly associated with active LBP symptoms and segmental instability, thus reflecting a state of active degeneration and biomechanical instability of the lumbar spine. Type 2 lesions are less clearly associated with LBP and appear to indicate a more biomechanically stable state, although stressors may cause them to reverse conversion into type 1 lesions. The exact nature and pathogenetic significance of Type 3 lesions remain largely unknown.

After completing therapy, the patients were re-examined orthopedically using the Cyriax method. The effect of the therapy was evaluated on the basis of pain reduction according to the visual analog scale (VAS), which was converted into percentages (%) in the statistics, where each grade of the VAS scale corresponds to a 10% improvement in spinal mobility in % with respect to the physiological norms of the age category, stretching tests (SLR and PKB), which included such elements as an improvement in range of motion and pain reduction during the test. The SLR (straight leg rise) is a test of the mobility of the dura mater of the spinal cord and nerve roots from L4 to S2. It involves performing a passive elevation of the lower limb to 90 degrees while lying on the back [21]. The PKB (prone knee bend) is the equivalent of the SLR for the L3 root. It involves passive knee flexion in the prone position. Both tests (SLR and PKP) are performed during the examination of the patient’s neurological status, taking advantage of the fact that the patient is lying in a supine or prone position [21]. The effect of the therapy was evaluated based on the reduction of pain (in percentages), improvement in spinal mobility (in percentages), and stretching tests (SLR and PKB), which considered elements such as the improvement in the range of motion and reduction of pain during the test. The evaluation of therapy outcomes did not include the increase in strength of weakened muscles, improvement of sensation and reflexes, or the presence of the Babinski sign, as patients with these symptoms were referred for further neurosurgical diagnostics before participating in the study.

### 2.5. Statistical Analysis

All data were analyzed using licensed software Statistica 13 (StatSoft, Inc. Tulsa, OK, USA) and R software (version 4.1.1, R Core Team (2021)). The Shapiro–Wilk test was used to evaluate the distribution of quantitative variables. Due to significant deviations from the normal distribution for most variables, non-parametric Mann–Whitney or Kruskal–Wallis tests were used in testing the statistical hypotheses for quantitative variables. Quantitative variables were characterized using the median and interquartile range (IQR). Spearman’s rank correlation coefficient (rho) was used to assess the strength of the relationship between quantitative variables. The chi-square test or Fisher’s exact test was used to analyze qualitative variables. Qualitative variables were characterized by the counts and percentages of each category.

## 3. Results

The basic demographic characteristics of the control and study groups, as well as the prevalence of disc pathologies, are shown in Table 1. There was no statistically significant difference regarding the age of the subjects in the control and study groups, which in the former case was 55 years, while in the latter it was 53 years. The control group was predominantly female, while the study group was male. The two groups did not differ in terms of the severity of degenerative disc disease as assessed by the degree of disc dehydration, the number of discs displaced intradurally, or the presence of canalicular tightness, myeloid degenerative reactions, and compression of spinal nerve roots.

**Figure 1:**
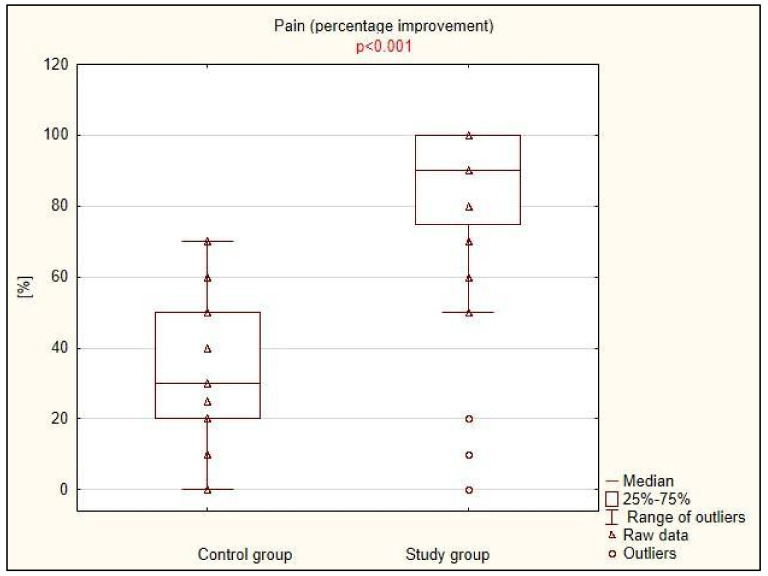
Clinical improvement in terms of pain in control and study groups.

**Figure 2:**
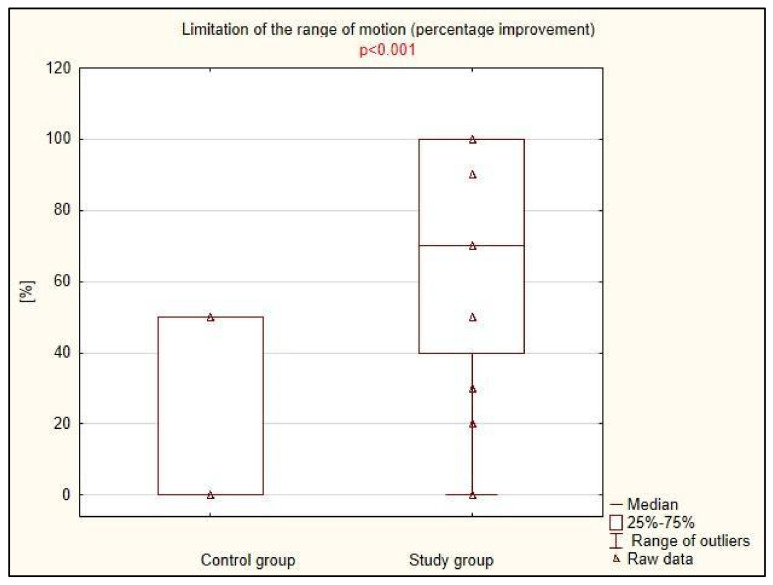
Clinical improvement in the range of motion in control and study groups.

**Figure 3:**
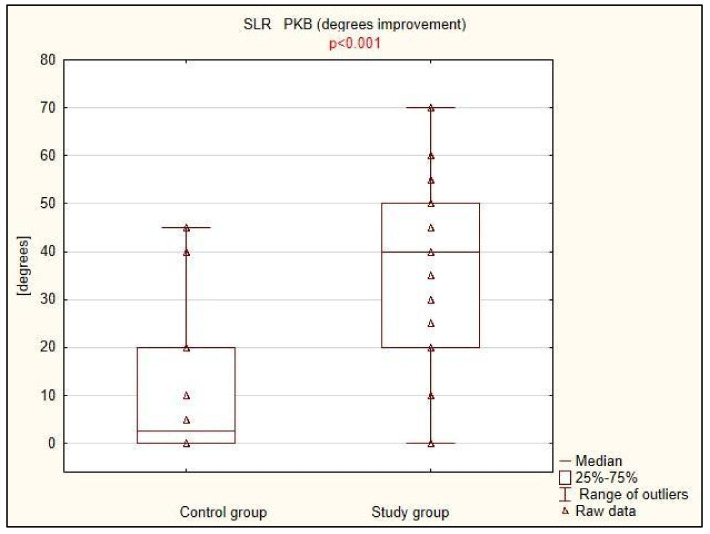
Clinical improvement in the stretch tests in control and study groups.

There was a statistically significant difference between the treatment effects assessed by the degree of pain and mobility limitation, as well as the presence of stretch symptoms: all three parameters improved significantly more in the study group compared to the control group (Table 2, Figure 1, Figure 2 and Figure 3). The difference was particularly striking regarding the limitation in the range of motion, which decreased by an average of 70% in 20 patients in the study group, while it did not improve in any of the 17 patients in the group treated with traditional physiotherapy methods.

After dividing the study group into three age subgroups (17–39 years, 40–59 years, and 60–79 years), there was no statistically significant gender differentiation or differences in the degree of disc disease, with the exception of the mean degree of dehydration, which was significantly lower in the youngest subgroup, where it was 1.6, while in the other subgroups it was 3.1 and 3.4, respectively (Table 3). The effects of Fascial Manipulation did not differ between the age subgroups (Table 3).

## 4. Discussion

Most publications on disc-related causes of lower back pain emphasize the great importance of intervertebral disc damage in generating lumbar pain. According to the American Academy of Orthopedic Surgeons, 80% of cases of low back pain are caused by disc herniation [22]. Buirski and Silberstein found that the more degenerated the intervertebral disc, the more likely it is to be responsible for the pain [23]. No abnormal signal pattern of the lumbar disc could be identified in MRI studies that would be indicated as a trigger for pain. Boos et al. showed that among symptomatic patients, almost all (96%) have damaged intervertebral discs, with 35% of the damage being extrusions [24]. In most other publications, the percentage of damaged discs as causes of lumbar pain is high, ranging from 17% to 62%, depending on the degree of intervertebral disc damage [25].

However, this is not to say that disc damage always causes pain, because between 25% and 85% of patients without pain symptoms have disc herniations [26]. For example, in a study by Jensen et al. 52% of asymptomatic patients had bulging, 27% had disc protrusion, 1% had extrusion, and 14% had a discontinuity of the outer annulus fibrosus [27]. According to Boos et al., in a group of patients without pain symptoms 76% had disc herniations of the lumbar spine, of which 13% were extrusions [24]. Another study on 302 women between the ages of 16 and 80 with no symptoms of disc damage found intervertebral disc abnormalities (mainly disc herniations) in 35% of patients under 40 years of age and in 50% of patients over 40 years of age [28]. Disc dehydration (mainly L4-5 AND L5-S1) occurred in about 25% of asymptomatic patients participating in the study described by Rapala et al. [29]. Therefore, many patients show no clear correlation between the presence of disc herniations of the lumbar spine confirmed by MRI and the presence of pain or symptoms that do not coincide with their clinical picture, characteristic of disc herniations.

Prior to this study, we hypothesized that the structure responsible for this phenomenon is the fascia. We assumed that disorders at the fascial level and all secondary disorders caused by them could cause pseudoradicular and other nonspecific pains in the lumbar spine. The problem of the failure to determine the cause of LBP affects 98% of patients in whom pain has first appeared and 90% of patients with recurring pain [30]. It is precisely these complaints—difficult to put into diagnostic and therapeutic schemes—that are known as nonspecific back pain [31].

In order to verify our hypothesis, we decided to subject the patients participating in this study to therapy using Fascial Manipulation. All patients had at least one disc herniation of the lumbar spine confirmed by MRI. This was an absolute condition to qualify patients for the study. Other abnormalities such as myelomeningocele, canal stenosis, and spinal root compression were not required, but were taken into account during the statistical analysis performed after the study was completed.

A marked improvement was observed in the study group compared to the control group in all parameters studied, i.e., reduced pain, increased range of motion, and better stretch test results (PKB and SLR), which indicates the high effectiveness of fascial therapy. The effectiveness of the therapy was not affected by age, gender, degree of intervertebral disc degeneration, or number of disc herniations, suggesting that it can be successfully applied to numerous patients regardless of the severity of degenerative spine disease.

We have found no articles with a similar research program in the available literature. The few papers evaluating Fascial Manipulation concern the treatment of nonspecific sacroiliac pain syndrome or other regions. They confirm the greater efficacy of FM over the traditionally used conservative treatment regimen for patients with lumbar disc disease, and show that the method has a great impact on improving musculoskeletal physiology.

The largest group of patients (102) was included in the study by Harper et al., who used the NPRS (Numerical Pain Rating Scale), GROC (15-point Global Rating of Change), and ODI (Oswestry Disability Index) to monitor progress. The NPRS scores in the FM-treated group increased threefold compared to the traditionally treated group (control). The increase in the number of patients whose GROC score increased by at least 5 points in the FM group was 92% vs. 45% in the control. The decrease in the ODI index by 10% was 96% vs. 70%. A 50% decrease in the ODI index was found in 65% patients vs. 40% in the control [32].

Brancini et al. randomized patients with chronic nonspecific lower back pain into two groups. One group was treated with FM and patients in the other group received a physiotherapy program according to the guidelines for CALBP (chronic nonspecific low back pain) with the inclusion of cupping therapy. Eight treatments were performed over a 4-week period. The patients’ condition was assessed using the VAS (Visual Analog Scale), BPI (Brief Pain Inventory), RMDQ (Rolland-Morris Disability Questionnaire), and SF-36 (well-being status using a brief health questionnaire). The examination of the aforementioned tests was performed before therapy, immediately after therapy, and 1 month and 3 months after the completion of therapy. Analysis of the results showed that there was a correlation between fascial thickness and lumbar pain. Treatment with FM led to a reduction in symptoms (especially pain) and improved function and well-being to a greater extent than with traditional treatment. By restoring fascial mobility proximal and distal to the site of pain, the range of motion increased and stiffness and pain were reduced [33].

A comparison of the effectiveness of FM and standard physiotherapy treatment in LBP was also made by Priya and Varghese, finding the former superior in reducing pain and improving range of motion [34].

Furthermore, in a study by Sawamura and Mikami, in which selected arm muscles were treated with FM, the Fascial Manipulation induced significant improvements in such muscle parameters as RT (muscle contraction reaction time), PMT (premotor time), MT (motor time), TPF (time to peak force), and TPA (time to peak activity) [35].

The significant efficacy of FM in reducing pain and improving range of motion is also highlighted by a meta-analysis including 10 papers on pain complaints in various locations [36]. The results of the statistical analysis may indicate that intervertebral disc damage may be a secondary effect of soft tissue pathology. Additionally, pain, in addition to cases of direct compression on highly sensory innervated structures such as the dura mater of the spinal cord or the meningeal sheath of the spinal root and the presence of inflammation accompanying disc damage, may result from soft tissue pathology such as ischemia and chemical changes within the fibrous connective tissue. These changes can be considered primary and may be caused by factors such as the disruption of muscle tone, impaired neuromuscular coordination, loss of fascial elasticity due to trauma, surgery or chronic tissue overload, or deterioration of the physical and chemical properties of the fascia due to poor diet, lack of physical activity, and unfavorable working conditions [15,37,38]. In studies by Hughes et al., it was described that Fascial Manipulation (FM) can be an effective therapeutic approach in cases of acute pain associated with fascial densification [39].

A limitation of the presented research program is the time of the Fascial Manipulations. Although standard therapy with FM lasts about four weeks, the duration of the therapy performed in this study was deliberately limited to two weeks for two reasons. First, this was to match the time in which patients in the study group were treated with the time of the standard treatment in the control group. Second, a longer period of treatment could suggest that the improvement in the patient’s condition was the result of self-healing.

Our results show that Fascial Manipulation is an effective method of treating patients with damaged intervertebral discs. At the same time, the lack of correlations between the number of damaged intervertebral discs, the degree of damage to the discs, and the effectiveness of fascial therapy indicated that in many cases it was not the disc damage but fascial disorders that were responsible for the patient’s complaints. This is confirmed by the fact that there are so many cases of differences between patients’ symptoms and signs and MRI images.

In pain research, the focus is mainly on the stimulation of peripheral nociceptors without determining the cause [39]. Meanwhile, the accumulation of HA (hyaluronic acid), a major component of GAG (glycosaminoglycan), can induce autoaggregation, causing a sharp increase in viscosity in the extracellular matrix. This change can affect polymodal nociceptors located in the deep fascia, lowering their threshold for activation [40,41,42,43]. This means that pain signals can be easily generated from “disturbed” tissues (e.g., the stiff fascia) and relayed by relay neurons in the posterior horns of the spinal cord to the brain [44]. Identifying the densification of specific areas of deep fascia can better guide therapy, contributing to a more nuanced view of the pain mechanism. The use of an innovative magnetic resonance imaging technique—T1ρ mapping—is very helpful in objectifying the progress of FM and in elucidating previously unknown pain mechanisms. It can also be helpful in the decision of selecting areas for FM therapy. A study by Menon et al. showed that high concentrations of unbound moisture were present to a greater extent in areas lying outside of symptom sites [45]. At the same time, these are the regions where FM therapy was performed. Increased T1ρ values reflect greater deposits of unbound GAGs and HA, which are therefore unable to express their “lubricating” properties within the deep fascia. This peculiar condition of isolated self-aggregating GAGs or HAs without water may explain the stiffness as well as the perceived pain caused by the irritation of free nerve endings and receptors within the deep fascia which do not have proper slippage between their layers or the underlying muscles [46,47].

Thus, T1ρ mapping suggests the usefulness of techniques that are performed in areas distant from the sites of pain. In addition, the significant effect of Fascial Manipulation may be related to other phenomena observed early on, when disc compression occurs in the first and second decades of life with upright posture and increased body weight, resulting in elevated tension in individual segments. Injuries, stresses, aggravating work patterns, and illnesses in the following decades cause tensions at further levels. All these events accumulate and cause disc misalignments.

In most patients’ MRI, disc problems most often appear at the L4-L5 and L5-S1 levels. However, in a large group of these patients, the discomfort and strains appear at the L2 level, since the greatest pressure is on the L2 level when standing and on the L4-5 level when sitting [48]. This happens because all the sympathetic nervous system fibers supplying the L3-5 segments reach the L2 spinal ganglion. This ganglion is positioned slightly more forward compared to ganglia at other levels and, in addition, in the standing position the L2 level receives the greatest load. The general rule of thumb is that the lower the segment, the greater the compression. This does not necessarily translate into the tensions on individual discs. Often, the release of tension in the higher segments also reduces the tension in the lower segments. Therefore, if a patient has symptoms at specific levels of the spine, a therapist should not just focus on them but also recognize where the tensions are highest. Releasing them will also cause relaxation in the segments where the patient was experiencing discomfort [49].

This phenomenon explains a significant percentage of diagnoses that disagree with MRI images (false positives or false negatives). Lumbar pain with damage to the intervertebral discs at the L4-5 levels causes tension at the L2 level, which, by irritating the lumbar plexus, can also result in pain in the lateral aspect of the hip plate, the hip joint, the side of the thigh or the area of the anterior superior iliac knee (MRI in patients with such signs often does not show disc herniations or shows them at levels from which other areas of the body are innervated).

Under physiological conditions, the nerve root is free inside the intervertebral foramen. If the tension on the disc increases, then there is too much pressure on this opening and the ligament of the Forestier (inside the intervertebral foramen), which impedes the mobility of the nerve root. In therapy, indirect (via the peripheral nerve) mobilization of the root is used, which has the effect of reducing the pressure inside the intervertebral disc. Such neuromobilization also aims to improve the elasticity of the meningo-vertebral ligaments (e.g., Hofmann’s ligaments), giving fibers to the posterior longitudinal ligament. These ligaments allow the inextensible dura mater to move (during trunk flexion up to 8–9 cm in the lumbar region) and are responsible for regulating pressure inside the spinal canal, while the lack of dura mater mobility manifests as pain in the back or lower limb [50,51].

In conclusion, the relatively low effectiveness of traditional conservative treatments is due to the focus on the patient’s symptoms and where they occur, rather than on the root causes, which are often located away from the lumbar spine [52,53,54].

### Limitations

This study is a series of observations from a single center, so it would be worthwhile to expand the results to include multi-center studies. A limitation of the presented research program is the duration of the Fascial Manipulation. Although standard therapy using FM lasts about four weeks, the therapy duration in the study described in this work was deliberately limited to two weeks for two reasons. First, this was intended to align the treatment time for patients in the study group with the standard conservative treatment time that patients in the control group underwent. Second, a longer therapy period could lead to doubts that the improvement in the patient’s condition might be the result of the self-healing processes previously described.

The existence of many factors that can influence the patient’s condition requires a holistic view of their body. Fascial Manipulation is a therapeutic method that treats the patient in this way. A characteristic feature of it is the simplicity of the techniques, which do not require great physical skill or strength from the therapist. However, knowledge of anatomy, biomechanics, and the physiology of the locomotor system is essential. In FM (Fascial Manipulation), the term “diagnosis” is not used; it is replaced with “hypothesis”. This indicates that the existence of many possible causes for the patient’s complaints is allowed from the outset. The hypothesis is corrected during palpatory verification, movement assessments, and therapy effectiveness evaluation. Therefore, in FM, the pre-therapeutic stage is the most important. Collecting important information during the interview, properly conducting palpatory verification, and performing movement tests facilitate the establishment of an accurate hypothesis. The measure of its accuracy is the effectiveness of the therapy performed. The lack of therapeutic effect is usually not a consequence of poorly conducted treatment but results from an incorrectly established hypothesis. Knowing that there may be many causes of pain or other dysfunctions in the patient, this is not considered a therapeutic error. Of course, in such a situation, conclusions should be drawn and another hypothesis should be formulated based on the concept of tensegrity. Therefore, when choosing CC and CF for therapy, one should not limit oneself to the area where pain symptoms occur.

## 5. Conclusions

Fascial Manipulation is an effective method for treating pain in patients with disc herniations of the lumbar spine; in addition to reducing pain, it improves the range of motion and the results of SLR and PKB tests. In lumbar spine pain in disc herniations, treatment using Fascial Manipulation is definitely more effective than treatment according to the traditional physiotherapy regimen.

## Figures and Tables

**Table 1:** Clinical characteristics of the control and study groups.

	Control (n = 33)	Study Group (n = 36)	*p*-Value
Age, Me (IQR)	55 (10) (n = 33)	51 (23) (n = 36)	0.179
Sex (K/M) n; (%)	24/9; (72.7/27.3)	15/21; (41.7/58.3)	0.015
Disc dehydration, Me (IQR)	3.2 (1.2)	2.4 (1.6)	0.080
Number of displacements, n	2.0	2.0	0.461
Canal stenosis (Yes/No), n; (%)	1/32; (3.0/97.0)	4/32; (11.1/88.9)	0.359
Myeloid reaction (Yes/No), n; (%)	6/27; (18.2/81.8)	13/23; (36.1/63.9)	0.113
Spinal nerve compression (Yes/No), n; (%)	5/28; (15.2/84.8)	10/26; (27.8/72.2)	0.251

Legend: Me—median, IQR—interquartile range.

**Table 2:** Clinical improvement in the study and control groups.

	Control Group (n = 33)	Study Group (n = 36)	*p* *
Pain (% improvement), Me (IQR)	30.0 (30.0)	90.0 (22.5)	<0.001
Limitation in the range of motion (% improvement), Me (IQR)	0 (50)	70 (55)	<0.001
SLR PKB (improvement), Me (IQR)	2.5 (20)	40 (30)	<0.001

Legend: Me—median, IQR—interquartile range. Notes: *—Mann–Whitney test or Fisher’s exact test.

**Table 3:** Characteristics and clinical improvement by age category in the study group.

	17–39 (n = 11)	40–59 (n = 12)	60–79 (n = 12)	*p* *
Sex (F/M) n; (%)	6/5 (54.5/45.5)	3/9 (25.0/75.0)	6/6 (50.0/50.0)	0.297
Dehydration, Me (IQR)	1.6 (0.3)	3.1 (1.2)	3.4 (2.2)	<0.001
Number of displacements n; (%)	1.0 (2.0)	2.0 (1.25)	2.0 (2.25)	0.391
Stenosis (Yes/No) n; (%)	1/10 (9.1/90.9)	1/11 (8.3/91.7)	2/10 (16.7/83.3)	0.779
Modic (Yes/No) n; (%)	2/9 (18.2/81.8)	4/8 (33.3/66.7)	7/5 (58.3/41.7)	0.130
Nerve compression (Yes/No) n; %	2/9 (18.2/81.8)	4/8 (33.3/66.7)	4/8 (33.3/66.7)	0.654
Pain (% improvement), Me (IQR)	90.0 (5.0)	100.0 (22.5)	80.0 (22.5)	0.068
Range of motion (% improvement), Me (IQR)	100 (0) (n = 2)	95 (35) (n = 8)	50 (50) (n = 9)	0.107
SLR PKB (improvement), Me (IQR)	45.0 (10.0) (n = 9)	40.0 (40.0) (n = 7)	32.5 (21.3) (n = 12)	0.290

Legend: Me—median, IQR—interquartile range, SLR-straight leg rise, PKB—prone knee bend. Notes: *—Kruskal–Wallis test or chi-square test.

## Data Availability

The data that support the findings of this study are available from the corresponding author upon reasonable request.
